# Waning of Anti-spike Antibodies in AZD1222 (ChAdOx1) Vaccinated Healthcare Providers: A Prospective Longitudinal Study

**DOI:** 10.7759/cureus.19879

**Published:** 2021-11-25

**Authors:** Sanjeeb K Mishra, Subrat K Pradhan, Sanghamitra Pati, Sumanta Sahu, Rajiv K Nanda

**Affiliations:** 1 Community Medicine, Veer Surendra Sai Institute of Medical Sciences And Research, Sambalpur, IND; 2 Field Epidemiology, Indian Council of Medical Research, Chennai, IND; 3 Biochemistry, Regional Medical Research Centre, Bhubaneswar, Bhubaneswar, IND; 4 Microbiology, Veer Surendra Sai Institute of Medical Sciences And Research, Sambalpur, IND; 5 Physiology, Veer Surendra Sai Institute of Medical Sciences And Research, Sambalpur, IND

**Keywords:** chadox1 ncov19 vaccine, astrazeneca vaccine, covishield, mixed model, kinetics, titer, antibody, covid vaccine

## Abstract

Introduction

Coronavirus disease 2019 (COVID-19) vaccines are nothing short of a miracle story halting the pandemic across the globe. Nearly half of the global population has received at least one dose. Nevertheless, antibody levels in vaccinated people have shown waning, and breakthrough infections have occurred. Our study aims to measure antibody kinetics following AZD1222 (ChAdOx1) vaccination six months after the second dose and the factors affecting the kinetics.

Materials and methods

We conducted a prospective longitudinal study monitoring for six months after the second of two AZD1222 (ChAdOx1) vaccine doses in healthcare professionals and healthcare facility employees at Veer Surendra Sai Institute of Medical Sciences and Research (included doctors, nurses, paramedical staff, security and sanitary workers, and students). Two 0.5-mL doses of the vaccine were administered intramuscularly, containing 5 x 10^10^ viral particles 28 to 30 days between doses. We collected blood samples one month after the first dose (Round 1), one month after the second dose (Round 2), and six months after the second dose (Round 3). We tested for immunoglobulin G (IgG) levels against the receptor-binding domain of the spike protein of severe acute respiratory syndrome coronavirus 2 (SARS-CoV-2) by chemiluminescence microparticle immunoassay. We conducted a linear mixed model analysis to study the antibody kinetics and influencing factors.

Results

Our study included 122 participants (mean age, 41.5 years; 66 men, 56 women). The geometric mean IgG titers were 138.01 binding antibody units (BAU)/mL in Round 1, 176.48 BAU/mL in Round 2, and 112.95 BAU/mL in Round 3. Seven participants showed seroreversion, and 11 had breakthrough infections. Eighty-six participants showed a substantial decline in antibody titer from Rounds 2 to 3. Persons aged 45 or older had higher mean titer than people aged younger than 45 years. Overweight and obese (BMI ≥ 25 kg/m^2^) had a higher mean titer than average or underweight persons. The only significant predictor of IgG titers at six months was SARS-CoV-2 infection on mixed model analysis.

Conclusion

We found a substantial decline in antibody levels leading to seven cases of seroreversion in healthcare professionals who received the ChAdOx1 vaccine. History of prior COVID-19 was the only significant factor in antibody levels at six months. Seroreversion and breakthrough infection warrant further research into the optimal timing and potential benefits of booster doses of the AZD1222 (ChAdOx1) COVID-19 vaccine.

## Introduction

Coronavirus disease 2019 (COVID-19) is caused by severe acute respiratory syndrome coronavirus 2 (SARS-CoV-2) and originated in China in late 2019. This zoonotic virus primarily affects the respiratory system and causes an illness similar to influenza, but in some cases, patients develop atypical pneumonia, respiratory failure, and approximately 2-3% of cases result in death [1].

COVID-19 is a health emergency on a scale not seen in several generations and the only practical way to disrupt the disease's progress in the community is through herd immunity [2]. Herd immunity is acquired when large portions of the population become immune to a specific disease by natural infection, vaccination, or both. Importantly, herd immunity protects medically vulnerable people who are unable to be vaccinated. Vaccine development is the most feasible means to achieve herd immunity in a population [3].

Early in the pandemic, the WHO recognized the potential of a vaccine to end the pandemic and started rolling out protocols for trials for vaccine candidates [4]. Seven vaccines received emergency use authorization (EUA) from the WHO, and many more are in development. Vaccine developers have tried a variety of mechanisms of action, including messenger ribonucleic acid (mRNA)-based vaccines, vector (adenovirus)-based vaccines, deoxyribonucleic acid-based vaccines, inactivated virus vaccines, and genetically modified virus vaccines [5].

In India, two vaccines were initially given EUA: a viral vector vaccine (AZD1222 (ChAdOx1)) manufactured by Serum Institute of India and developed by AstraZeneca in collaboration with Oxford University, and Covaxin (BBV152), an inactivated vaccine developed by Bharat Biotech and Indian Council of Medical Research [6]. Recently, the Sputnik V vaccine by Gamaleya Research Institute of Russia also received EUA. Of these three vaccine options, ChAdOx1 represents more than 88% of the vaccines administered in India [7].

ChAdOx1 was initially authorized as a two-dose regimen with a gap of 21 to 28 days. The gap was subsequently extended to 42 to 56 days, then increased to 84 to 112 days. Recently, India achieved one billion doses of administered vaccine, accounting for nearly 50% of the adult population in India, of which approximately 30% of the population have been fully vaccinated as of this writing [7].

Many studies have highlighted the dynamics of seroconversion following natural infection. Coronaviruses encode four major structural proteins (spike, membrane, envelope, and nucleocapsid) [8]. Antibodies against spike protein's receptor-binding domain (RBD) have been demonstrated as neutralizing in most studies; therefore, most vaccines target the spike protein RBD and induce immunoglobulin G (IgG) against it [9,10].

Seroconversion with IgG for most patients takes place within seven to 14 days, and the concentration of these antibodies reaches a peak value approximately one month (21 to 49 days) after infection. These neutralizing antibodies persist for up to four months post-infection [11]. The antibody response differs significantly in patients with mild and severe forms [11,12]. Asymptomatic patients had lower antibody titers than symptomatic individuals; as many as 87% of the asymptomatic COVID-19 patients did not develop detectable antibody levels, highlighting the transient nature of IgG levels following COVID-19 infection.

The dynamics of IgG following vaccination are still being studied. Some studies have reported waning of antibody levels at six months after the second vaccine dose [13]. However, other studies have highlighted that a significant amount of IgG persists at six months following the second dose [14,15]. The seroprotection level and correlates of protection have not yet been established [15]. The knowledge of the required antibody levels and their dynamics are the basis for further research to better understand the protective mechanisms, pathogenesis, and prognostic factors of COVID-19. This will also be key in the development of effective treatments and vaccines. While many studies on the mRNA vaccines exist, studies on the trends of antibodies following the ChAdOx1 vaccine remain limited. Therefore, we conducted this study to evaluate the antibody kinetics following AZD1222 (ChAdOx1) vaccination up to six months following the second dose and the factors affecting the kinetics.

## Materials and methods

Study design and setting

The healthcare providers and employees of Veer Surendra Sai Institute of Medical Sciences and Research (VIMSAR) comprised the study population and included doctors, nurses, paramedical staff, security and sanitary workers, and students. The study was approved by State Research and Ethics Committee, Odisha (approval 3216/11-02-21) and it was conducted according to the declaration of Helsinki and WHO Good Clinical Practices. The participants were informed in the local language about the study, and they provided written consent prior to enrollment. We conducted a prospective longitudinal study with a cohort of 122 participants who consented to be a part of the study amongst the vaccinated healthcare providers of VIMSAR. We organized participants into four groups: doctors, nurses, students, and others (which included paramedics, security, and sanitary workers).

Two 0.5-mL doses of the ChAdOx1 vaccine were administered intramuscularly 28 to 30 days apart, each dose containing 5 x 1010 viral particles. Blood samples were collected one month (28 to 33 days) after the first dose (i.e., Round 1), one month (28 to 32 days) after the second dose (i.e., Round 2), and six months (180 to 192 days) after the second dose (i.e., Round 3). The data were recorded in a predesigned questionnaire using the interview method. We recorded sociodemographic variables including age (as age 18 to 44 years or 45 and older), sex, educational level, occupation, and blood group. The questionnaire included comorbidities such as diabetes and hypertension. We recorded participants' histories of previous COVID-19 status diagnosed either by rapid antigen testing, real-time polymerase chain reaction (PCR), or Truenat® SARS CoV-2. chip-based real-time micro-PCR test (Molbio Diagnostics Pvt. Ltd., Verna, Goa). We also measured participant height, weight, and blood pressure. Our dependent variable was the anti-spike IgG antibody titer.

After collecting 3 mL blood by aseptic procedure, we spun the samples via centrifuge at 2500 rpm for five minutes. The separated serum was transferred to a cryogenic vial (2 ml) and was stored at -20°C. The samples were transported at 2°C to 8°C to the serology laboratory.

Test principles

We conducted quantitative detection of antibodies against SARS-CoV-2 spike protein in chemiluminescence microparticle immunoassay. This method used the principle of double-antigen sandwich assay and provided results within 18 minutes.

Laboratory assay was completed using the Abbott Architect i2000SR immunoassay analyzer (Abbott Laboratories, Abbott Park, Illinois) following the manufacturer's package insert for the SARS-CoV-2 IgG II Quant assay [16]. It is used for the qualitative and quantitative determination of IgG antibodies to the RBD of the S1 subunit of the spike protein of SARS-CoV-2 in human serum and plasma. The sequence used for the RBD was from the WH-Human 1 coronavirus. The manufacturer-defined analytical measurement interval is 21 to 40,000 AU/mL, and the positivity cut-off is ≥50 AU/mL. This method has a sensitivity of 91.6% at all time points and 98.3% at >14 days; its specificity is 99.4% [16]. This method showed high concordance with the neutralizing antibody titer level with very high specificity [17]. The WHO's international standard units for binding antibodies (i.e., BAU/mL) are reported in this article for assays to allow comparisons across different platforms and studies. The manufacturer-defined multiplication factor of 0.142 to the value in AU/mL was used in the calculation [18].

Statistical analysis

As the dataset was highly skewed, it was transformed with log 10. We performed descriptive analysis and linear mixed model analysis after log transformation of antibody titer levels. Geometric mean and median were used as measures of central tendency, and confidence interval (CI) and interquartile range (IQR) depicted the spread.

We used linear mixed models to examine the antibody kinetics six months after participants received the second vaccine dose and to associate these changes with the participants' demographic characteristics and coexisting conditions. The dependent variable was the antibody level, which was log-transformed. Fixed effect covariates included sex, age group (18 to 44 years and 45 to 64 years), age by sex interaction, and random factors included individual regression intercepts. Slopes from individual participants were modeled using maximum likelihood methods. Unstructured covariance structure was selected in building the model to fit the data based on -2log likelihood value. Multiple comparisons were made after the Bonferroni adjustment. There were no missing data. A two-sided p-value < 0.05 was considered statistically significant. Error bar graphs of log-transformed antibody levels are drawn using GraphPad Prism Version 8.02 (Released February 6, 2019, GraphPad Software Inc., San Diego, California), and linear mixed model analyses were performed using IBM SPSS Statistics for Windows, Version 25.0. (Released 2017, IBM Corp., Armonk, New York). We checked residuals for normality.

## Results

Our study cohort included 122 participants. The characteristics of the participants are shown in Table 1. The median age of the participants was 41.5 years (range 18 to 65 years). Sixty-six participants (54%) were younger than age 45, 56 (46%) were aged 45 or older. Sixty-six participants (54%) were men, 56 were women (46%). Most participants were in the "doctor" or "others" groups, representing 61% of the total. Twenty-two had hypertension and 18 had diabetes. Sixty-eight participants (55.7%) had a BMI < 25 kg/m2 and 31 (25%) had a history of COVID-19 infection with 11 breakthrough infections (14 days after the second dose).

**Table 1:** Characteristics of participants and GMT of antibody level according to group and time (N=122) COVID-19: coronavirus disease 2019; GMT: geometric mean titer; N: number; BAU: binding antibody units

Variables	Antibody GMT (BAU/mL) one month after first dose (95% CI)	Antibody GMT (BAU/mL) one month after second dose (95% CI)	Antibody GMT (BAU/mL) six months after second dose (95% CI)
Age category			
<45 years (n=66)	89.41 (45.17, 176.98)	173.24 (130.71, 229.86)	103.44 (66.36, 158.56)
≥45 years (n=56)	223.31 (126.55, 394.05)	180.82 (120.12, 270.69)	125.52 (74.20, 211.39)
Sex			
Male (n=66)	164.46 (118.39, 353.06)	169.71 (115.74, 249.42)	141.34 (87.38, 228.06)
Female (n=56)	84.26 (39.91, 177.86)	185.17 (142.96, 240.92)	86.35 (63.32, 118.40)
Occupation			
Doctor (n=36)	133.41 (60.56, 293.89)	138.22 (88.50, 215.89)	97.09 (47.69, 197.64)
Staff nurse (n=25)	135.18 (56.76, 321.92)	165.27 (104.39, 261.59)	110.35 (52.56, 229.63)
Student (n=24)	43.16 (11.73, 158.87)	224.79 (135.45, 373.89)	103.97 (52.78, 205.97)
Other (n=37)	193.63 (141.47, 409.46)	199.05 (127.58, 313.48)	140.60 (80.95, 244.75)
BMI			
<25 (n=68)	100 (52.72, 189.66)	171.70 (126.44, 231.81)	111.32 (75.94, 166.46)
≥25 (n=54)	180.63 (105.91, 380.08)	183.03 (123.03, 270.39)	114.64 (64.91, 201.12)
Diabetes			
No (n=104)	120.23 (73.67, 196.20)	165.13 (128.71, 211.980	109.46 (76.86, 158.08)
Yes (n=18)	248.68 (79.66, 974.86)	257.68 (120.05, 551.09)	131.32 (55.33, 310.40)
Hypertension			
No (n=100)	124.37 (74.55, 207.46)	172.71 (132.09, 226.47)	109.48 (75.83, 157.17)
Yes (n=22)	185.12 (73.94, 568.98)	192.83 (111.67, 333.42)	131.91 (57.99, 297.75)
COVID-19 Infection			
No (n=91)	114.18 (66.58, 195.83)	160.46 (124.96, 205.85)	76.53 (52.72, 110.20)
Yes (n=31)	220.82 (97.77, 530.86)	234.86 (129.59, 424.68)	356.04 (197.79, 645.12)

Seroconversion and seroreversion

All but one participant achieved seroconversion after the second dose of vaccine, representing a 99.2% seroconversion rate. Of 122 participants, 11 did not seroconvert one month after the first dose of vaccine; and one did not seroconvert even after the second dose (Round 2). Seven participants (three men, four women; O+ or B+ blood groups) showed seroreversion six months after the second dose. Five of them had a BMI > 25 kg/m2. One had a history of COVID-19 infection six months prior to vaccination.

Breakthrough infection

Eleven breakthrough infections took place over the six-month monitoring period (seven men, four women). Six breakthrough infections occurred in doctors. One was a case of reinfection (nine months from the first infection). All the breakthrough cases belong to either O+ or B+ blood groups.

IgG titer and kinetics

The geometric mean IgG titer was 138.01 BAU/mL (95% CI: 87.37, 217.99 BAU/mL) in Round 1, 176.48 BAU/mL (95% CI: 138.80, 224.39 BAU/mL) in Round 2, and 112.95 BAU/mL (95% CI: 80.97, 157.56 BAU/mL) in Round 3. The geometric mean in the 11 participants with breakthrough infection was 1073.86 BAU/mL (95% CI: 616.85, 1868.98 BAU/mL). In persons with prior history of COVID-19 infection, the geometric mean IgG titer was 194.68 BAU/mL.

The IgG titer increased from Round 1 to Round 2 for 88 participants. The 34 participants (27.8%) with a decrease in titer value had the maximum possible reportable titer at Round 1, and 12 of them (35%) had a history of COVID-19. Eighty-six participants (70%) exhibited a decline in their antibody titer six months from their peak in Round 2. They showed an average 72.7% decline in titer from Round 2 to Round 3. In the remaining 36 participants, the IgG level plateaued or increased; 11 were breakthrough cases.

The rise in antibody titer after the second dose of vaccine (Round 2) was highest among students (5.21 times), followed by nurses (1.82 times), doctors (1.03 times), and others (1.03 times). The decay in antibody levels from Round 2 to Round 3 (six months) was also highest for students (53%). Women showed a 2.19-fold rise in titer from Round 1 to Round 2 compared to a 1.03-fold rise for men. Women exhibited faster decay at 53% compared to 12% for men. Participants with diabetes and hypertension had a lower mean increase in titer from Round 1 to Round 2 than those without those conditions. Nevertheless, the geometric mean titer was higher for participants with hypertension and diabetes than those without these comorbidities at any given time point. Also, persons with a BMI >25 kg/m2 and persons older than 44 had higher mean titer values at all time points. We observed a decline in titer value in persons aged 45 and older from Round 1 to Round 2; 34% of them had the maximum possible titer value at Round 1.

**Figure 1:**
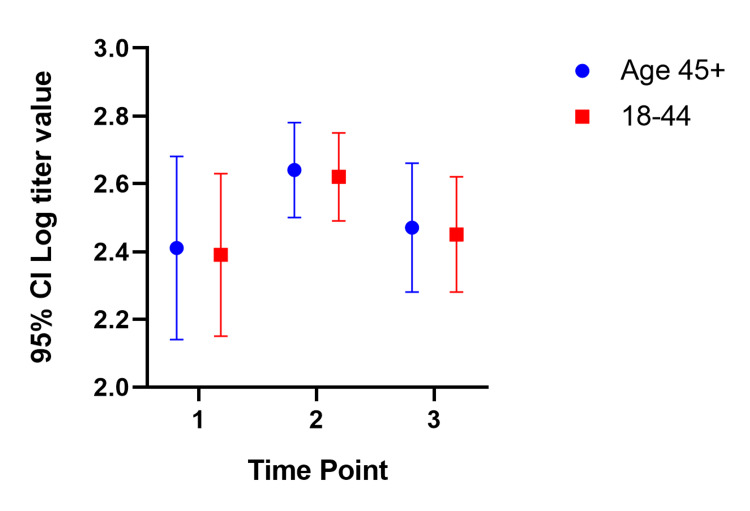
Mean (95% CI) and standard error of log-transformed antibody titer by time and age group. Time Point 1 = one month after first dose; Time Point 2 = one month after second dose; Time Point 3 = six months after second dose.

**Figure 2:**
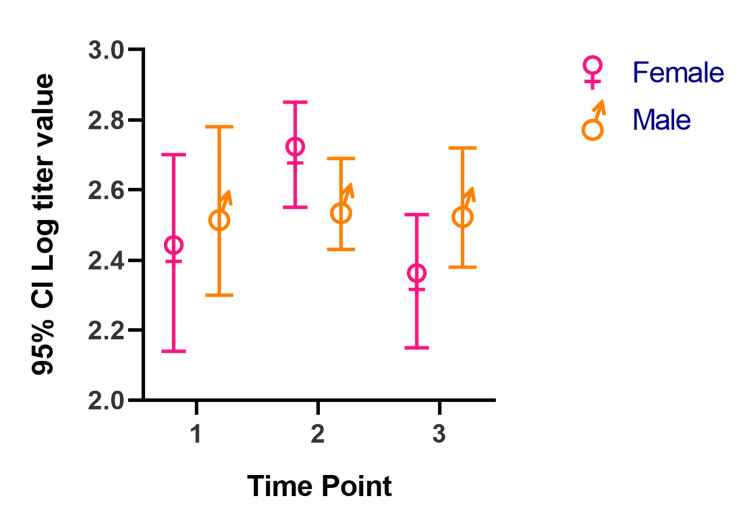
Mean (95% CI) and standard error of log-transformed antibody titer by time and sex. Time Point 1 = one month after first dose; Time Point 2 = one month after second dose; Time Point 3 = six months after second dose.

**Figure 3:**
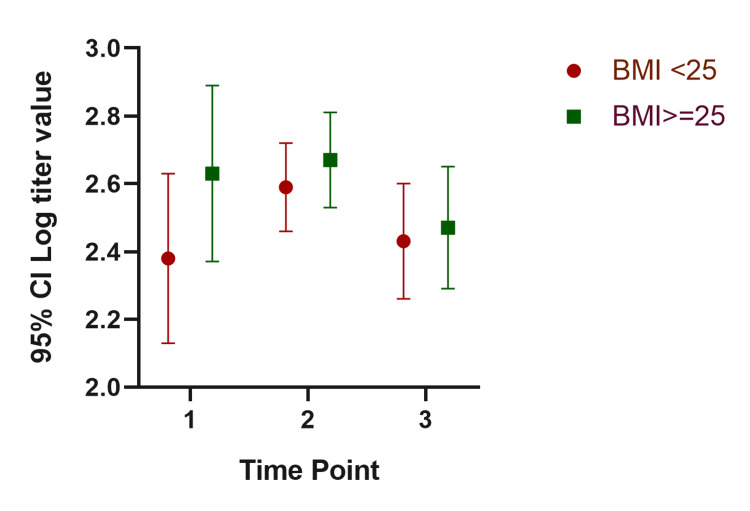
Mean (95% CI) and standard error of log-transformed antibody titer by time and BMI. Time Point 1 = one month after first dose; Time Point 2 = one month after second dose; Time Point 3 = six months after second dose.

**Figure 4:**
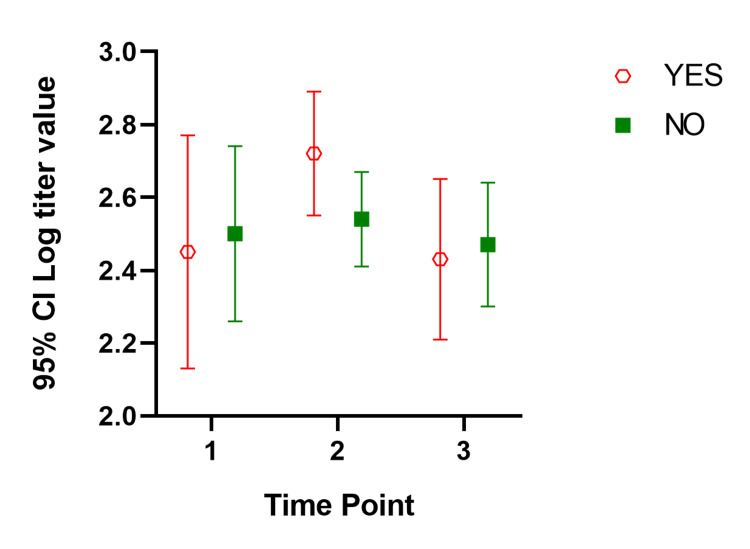
Mean (95% CI) and standard error of log-transformed antibody titer by time and diabetes status. YES indicates participants with diabetes; NO indicates participants without diabetes. Time Point 1 = one month after first dose; Time Point 2 = one month after second dose; Time Point 3 = six months after second dose.

**Figure 5:**
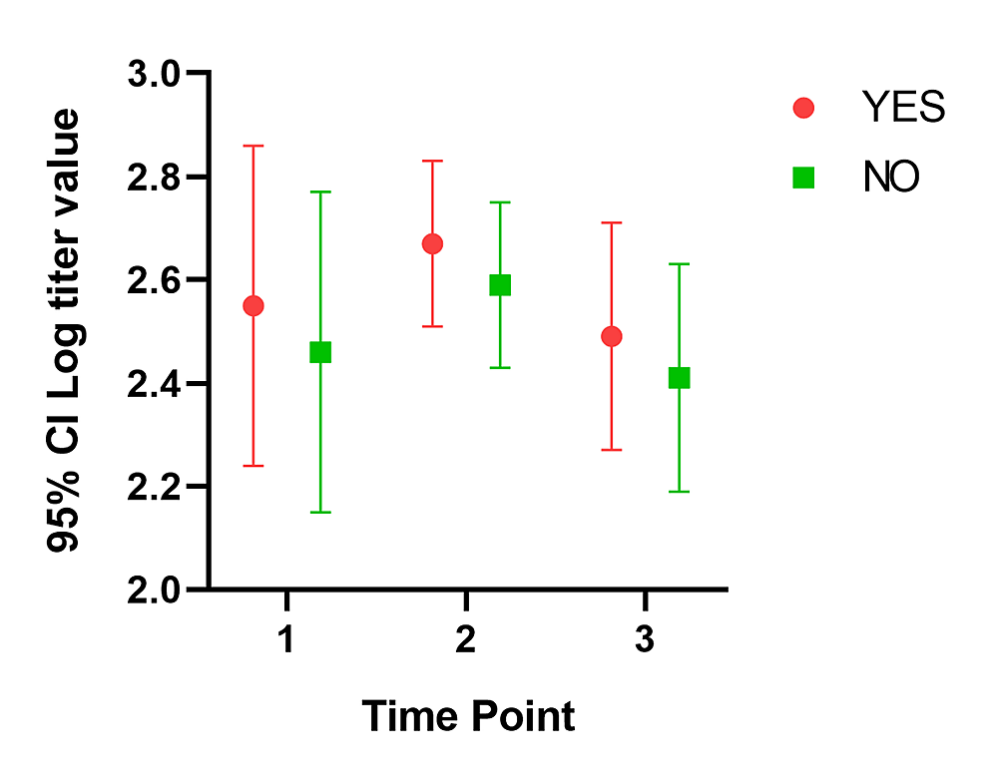
Mean (95% CI) and standard error of log-transformed antibody titer by time and hypertension status. YES indicates participants with hypertension; NO indicates participants without hypertension. Time Point 1 = one month after first dose; Time Point 2 = one month after second dose; Time Point 3 = six months after second dose.

**Figure 6:**
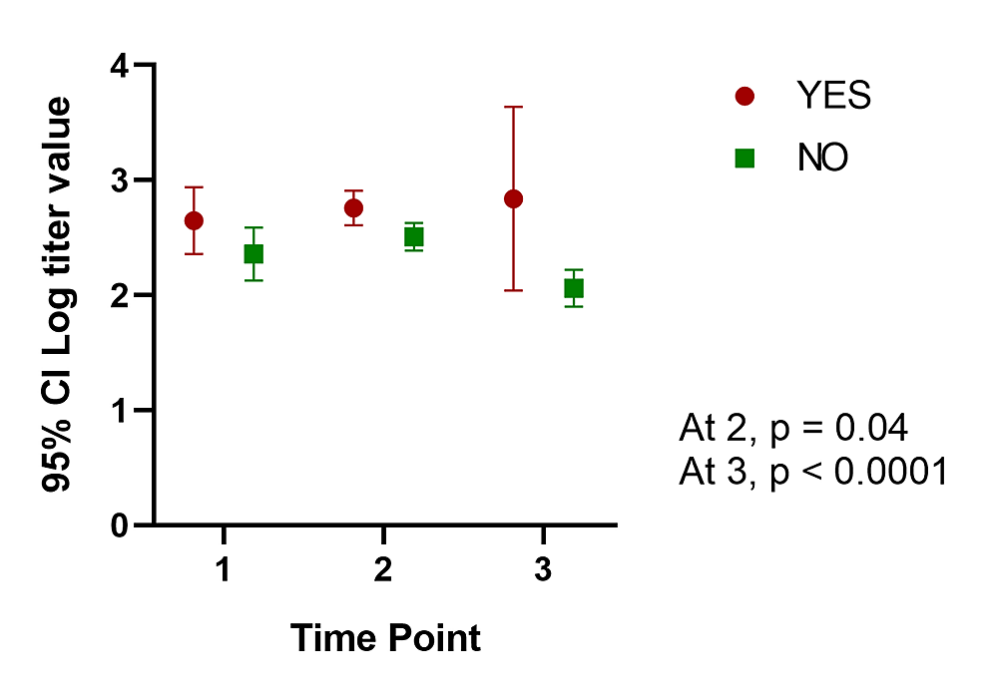
Mean (95% CI) and standard error of log-transformed antibody titer by time and history of COVID-19. Time Point 1 = one month after first dose; Time Point 2 = one month after second dose; Time Point 3 = six months after second dose. COVID-19: coronavirus disease 2019

In our mixed model, the mean titer values are not very different between groups at all the time points, and the considerable overlap in mean difference of 95% CIs shows that the means are not significantly different except for those with a history of COVID-19. Participants with a history of COVID-19 infection have significantly different mean titer values at one and six months after the second dose. The mean titer value in all groups increased one month after the second dose and decreased six months later. Figure 1, Figure 2, Figure 3, Figure 4, Figure 5, Figure 6 show error bar graphs (95% CI) of log-transformed antibody titer by time and groups.

Table 2 shows the mean difference between the groups for different variables at each time point. We found no significant difference between groups at all three time points except for the participants with a history of COVID-19. The mean difference between participants with a history of COVID-19 and those without was statistically significant one month after the second dose (mean difference: -0.25; 95% CI: -0.5, -0.01; P=0.04) and six months later (mean difference: -0.78, 95% CI: -1.11, -0.4; P<.0001).

**Table 2:** Mixed-model analysis of variables associated with antibody titer after vaccination (N=122) COVID-19: coronavirus disease 2019; SE: standard error; N: number

Time Point Variable	Antibody Titer Level (Log-Transformed) Mean (SE)		Mean Difference (95% CI)	P-value
	Age			
	<45 years (n=66)	≥45 years (n=56)		
One month after first dose	2.41 (0.27)	2.39 (0.24)	-0.18 (-0.64, 0.28)	0.44
One month after second dose	2.64 (0.14)	2.62 (0.13)	0.008 (-0.24, 0.25)	0.95
Six months after second dose	2.47 (0.19)	2.45 (0.17)	0.007 (-0.33, 0.31)	0.96
	Sex			
	Female (n=56)	Male (n=66)		
One month after first dose	2.42 (0.28)	2.54 (0.24)	-0.17 (-0.65, 0.31)	0.48
One month after second dose	2.70 (0.15)	2.56 (0.13)	0.14 (-0.12, 0.39)	0.28
Six months after second dose	2.34 (0.19)	2.55 (0.17)	-0.21 (-0.55, 0.12)	0.20
	BMI			
	<25 kg/m^2 ^(n=68)	≥25 kg/m^2^ (n=54)		
One month after first dose	2.38 (0.25)	2.63 (0.26)	-0.25 (-0.67, 0.16)	0.26
One month after second dose	2.59 (0.13)	2.67 (0.14)	-0.08 (-0.29, 0.14)	0.49
Six months after second dose	2.43 (0.17)	2.47 (0.18)	-0.04 (-0.32, 0.25)	0.81
	Diabetes			
	No (n=104)	Yes (n=18)		
One month after first dose	2.50 (0.24)	2.45 (0.32)	-0.11 (-0.54, 0.75)	0.75
One month after second dose	2.54 (0.13)	2.72 (0.17)	-0.19 (-0.53, 0.15)	0.28
Six months after second dose	2.47 (0.17)	2.43 (0.22)	0.04 (-0.41, 0.49)	0.86
	Hypertension			
	No (n=100)	Yes (n=22)		
One month after first dose	2.46 (0.23)	2.55 (0.31)	-0.08 (-0.65, 0.49)	0.78
One month after second dose	2.59 (0.12)	2.67 (0.16)	-0.08 (-0.38, 0.23)	0.61
Six months after second dose	2.41 (0.16)	2.49 (0.22)	-0.08 (-0.48, 0.32)	0.69
	COVID-19 Infection			
	No (n=91)	Yes (n=31)		
One month after first dose	2.36 (0.23)	2.65 (0.29)	-0.29 (-0.76, 0.16)	0.22
One month after second dose	2.51 (0.12)	2.76 (0.15)	-0.25 (-0.5, -0.01)	0.04
Six months after second dose	2.06 (0.16)	2.84 (0.80)	-0.78 (-1.11, -0.46)	< .0001>

## Discussion

We found a substantial decline in levels of IgG directed towards the RBD of the spike protein S1 subunit of SARS-CoV-2 from peak values one month after the second dose of the ChAdOx1 COVID-19 vaccine. We noted a sharper decay rate of 72% compared to the 29% decay rate reported for people receiving the BNT162b2 vaccine and the 17% decay rate for people receiving the mRNA-1273 vaccine [19].

In 36 cases, the titer value increased from Round 2 to Round 3; 11 were confirmed breakthrough infection cases, and the rest may represent asymptomatic COVID-19 infection. This finding aligns with the report by Wei et al., who found a 95% reduction in symptomatic cases after two doses of vaccine and a 28% protection against asymptomatic infection [20]. Similar results were also seen in Tré-Hardy et al., where 9% of the participants showed an increase in titer from their value one month after the second dose [21]. In our study, 34 participants showed a decline in antibody titer from Round 1 to Round 2; all had maximum reportable titer value at Round 1, indicating prior SARS-CoV-2 infection. Twelve participants had documented prior history of COVID-19. Prior studies have indicated a large number of unreported asymptomatic infections during the COVID-19 pandemic [22].

In 70% of participants with a history of COVID-19, the second dose did not boost their IgG level. The lack of increase in IgG titer is due to the robust antibody response after a single dose of COVID-19 vaccine in people previously infected [20,23]. Like previous recommendations, we suggest that the second dose could be delayed for previously infected persons in this resource-limited setting, and other vulnerable groups should be prioritized [20].

We found higher mean titer values for persons aged 45 or older than 18-45 years, which contrasts with the findings in other studies [13,20,23]; however, the association was not statistically significant. A higher mean titer value was also seen in persons with either hypertension or diabetes but was not significant at a 5% level compared to other reports [13,20,24]. Persons with a BMI ≥25 kg/m2 showed higher mean titer than those with a BMI <25 kg/m2; similar results were shown in the previous study [15] but in contrast to the study by Pellini et al. [24].

ABO blood group has been implicated as a risk factor to infection and disease severity in COVID-19 [25-27]. Our study found that the seroreversion cases and breakthrough cases all had either B+ or O+ blood groups. Similar results of higher positive testing for B+ unvaccinated individuals were shown in the study by Latz et al. [27]. This risk of seroreversion and breakthrough infection in B+ and O+ individuals warrants further large-scale studies.

In a mixed-model analysis, only prior COVID-19 infection was a significant predictor of IgG titers after full vaccination at Round 2 and Round 3. We found no significant association for age, sex, BMI, or comorbidities, like Massarweh et al. [28]. However, another study on a larger scale reported a significant association for age, sex, and higher BMI [13].

Seven of the 122 participants had seroreversion at six months. Feng et al. demonstrated that the RBD IgG level of 165 BAU/mL one month after the second dose correlates with 80% vaccine effectiveness or seroprotection [15]. In this context, 62 participants in our study had a lower titer than the estimated seroprotection level at that stage. Nevertheless, studies have highlighted strong T-cell response and cell-mediated immunity following the ChAdOx1 vaccine administration; this may also contribute to overall protection [29]. However, in general, antibody titers have been directly linked with protection from symptomatic COVID-19 [30]; these seroreversion cases and persons with lower titers are likely to be at higher risk, necessitating a booster dose of the vaccine.

Limitations

Our study was limited in that weekly or monthly estimation of IgG titer would have been better for depicting antibody kinetics. However, given our resource-limited setting, we included three time points to increase compliance and avoid loss to follow-up.

## Conclusions

Our study demonstrates the high effectiveness of the AZD1222 (ChAdOx1) vaccine in achieving seroconversion one month after the second dose. Most patients showed substantial waning in IgG titer from Round 2 to Round 3. Cases of seroreversion and breakthrough are a concern requiring further evidence for a booster dose for high-risk health professionals. The association of blood group with seroreversion and breakthrough infection needs further investigation.
